# MoO_3_ Nanobelt-Modified HMCM-49 Zeolite with Enhanced Dispersion of Mo Species and Catalytic Performance for Methane Dehydro-Aromatization

**DOI:** 10.3390/molecules27144404

**Published:** 2022-07-09

**Authors:** Jing Hu, Yangyang Li, Shujie Wu, Xiaohui Wang, Cai Xia, Xinyu Zhao, Jinglin Liu

**Affiliations:** 1 Inner Mongolia Key Laboratory of Carbon Nanomaterials, College of Chemistry and Materials Science, Inner Mongolia Minzu University, Tongliao 028000, China; 15114714255@163.com (Y.L.); wxh1009@126.com (X.W.); 15560587095@163.com (C.X.); xyzhao@imun.edu.cn (X.Z.); 2 Institute of Physical Chemistry, College of Chemistry, Jilin University, Changchun 130023, China; shujiewu@2022126.com

**Keywords:** methane dehydro-aromatization, MoO_3_ nanobelts, MCM-49

## Abstract

The methane dehydro-aromatization reaction (MDA) is a promising methane valorization process due to the conversion of methane to value-added aromatics (benzene, toluene and naphthalene). However, one of the major disadvantages of utilizing zeolite in MDA is that the catalyst is rapidly inactivated due to coke formation, which eventually causes the activity and aromatic selectivity to decrease. Consequently, the process is not conducive to large-scale industrial applications. The reasonable control of Mo site distribution on the zeolite surface is the key factor for partially inhibiting the coking of the catalyst and improving stability. Here, MoO_3_ nanobelts can be used for alternative Mo precursors to prepare MDA catalysts. Catalysts modified with MoO_3_ nanobelts present higher activity (13.4%) and benzene yield (9.2%) than those catalysts loaded with commercial MoO_3_.

## 1. Introduction

Natural gas is a promising alternative to oil as an industrial carbon source. Methane is a clean and environmentally friendly fossil fuel because it mainly emits CO_2_ and water vapor after burning [1]. In addition, natural gas is usually transported by pipelines or in the form of liquefied natural gas. However, the construction of pipelines is extremely expensive and there is a risk of leakage. Consequently, there is a need to explore an efficient method for methane conversion into value-added chemicals from the perspective of methane utilization and environmental friendliness. MDA is one of the most attractive reactions, because the products are high-value petrochemicals (benzene, toluene and xylene) that can be applied to produce industrial raw materials [2].

Since Wang et al. reported the pioneering work that Mo/HZSM-5 catalyst can effectively enhance the catalytic performance, particularly the stability in MDA for the commercial viability of the process [3], there has been a great deal of research indicating that HZSM-5 zeolite is an excellent catalyst to provide a shape-selective condition for the conversion of methane to benzene. However, MDA reaction is a highly endothermic process: CH_4_ → C_6_H_6_ + H_2_, ∆_r_H_m_ = 532 KJ∙mol^−1^. Strong heat of adsorption means that higher benzene yields can be obtained at higher temperatures. The temperatures in the 300–1100 °C range are thermodynamically more inclined to produce graphite carbon (coke) [4,5]. Therefore, the efficient MDA catalyst should have the following conditions [6]: (i) the catalysts effectively activate the stable C-H bond in the CH_4_ molecule; (ii) the prepared materials selectively produce aromatics and minimize carbon deposition; and (iii) the catalysts have good stability at high temperature.

It is generally believed that the dispersion of metal Mo species in zeolite plays an important role in catalyst stability [7]. Many strategies have revealed improvements, including the addition of accelerators [2,8,9], modulation of the pH value of the impregnation solution [10], and modification of the zeolite surface by desilication/dealumination or silicification [11] to enhance the dispersity of Mo species. Furthermore, many alternative zeolitic structures have been developed and investigated with the aim of reducing carbon deposition at the active site in methane non-oxidative aromatization. Various zeolite carriers such as ZSM-5 [12], ITQ-13 [13], MCM-22 [14], MCM-49 [15], IM-5 [16] and TNU-9 [17] zeolites have been tested. The results show that HZSM-5 and MCM series zeolite remain effective carriers for promoting MDA activity. HMCM-49 exhibits higher stability and benzene selectivity than HZSM-5; HMCM-49 has a unique pore structure design with two independent pore systems: a two-dimensional 10-membered ring sinusoidal pore system (4.1 × 5.9 Å) and a larger three-dimensional 12-membered ring super cage system (7.1 × 7.1 × 9.1 Å). The two systems are interconnected by a 10-membered ring channel (4.1 × 5.4 Å). The type of pore structure and the existence of super cages may be beneficial for the high carbon accumulation capacity in HMCM-49 zeolite [18] and maintaining the selectivity to aromatics [19].

Nanostructured MoO_3_ materials have attracted extensive attention in photoelectric materials [20], heterogeneous catalysis [21,22], biochemical sensing [23], photothermal therapy [24], and other aspects because of its unique physical and chemical properties. The Ce-doped nano-MoO_3_ modification of ZSM-5 catalyst shows excellent SCR performance [25], the ordered mesoporous α-MoO_3_ nanocrystalline applied to thin film pseudocapacitors exhibits excellent capacitive charge storage performance [26]. Therefore, this study explores the use of MoO_3_ nanobelts as the precursor for Mo-based HMCM-49 to induce better dispersion of the active metal MoO_x_ in the pore channel of zeolite, thus effectively improving MDA catalytic performance.

## 2. Materials and Methods

### 2.1. Synthesis of MoO_3_ Nanobelts

First, 1 g (NH_4_)_6_Mo_7_O_24_·4H_2_O and 0.08 g citric acid were added to a 100 mL beaker, and 10 mL of deionized water was introduced and stirred thoroughly to fully dissolve the solid. Then, the pH value was regulated to 1 with 3 mol/L HCl solution; the mixture was ultrasonicated for 30 min and then transferred to a Teflon-lined stainless autoclave under static crystallization at 180 °C for 2 d. The reaction product was cooled to room temperature, vacuum pumped and filtered, and washed with anhydrous ethanol and deionized water several times. The obtained products were dried for 30 min in the drying oven at 100 °C, and blue powder products were obtained.

### 2.2. Preparation of Na-MCM-49 Zeolite

MCM-49 zeolite was fabricated by hydrothermal synthesis by applying hexamethylenediamine (HMI) as a hard template and silica sol as a silicon source. Firstly, a mixture of HMI, water, aluminum nitrate, sodium hydroxide and silica sol in a certain proportion was stirred for 12 h. The mole ratio of gel composition is SiO_2_:0.04Al_2_O_3_: 0.12NaOH:0.35HMI: 25H_2_O, and the mixture was transferred to a Teflon-lined stainless autoclave under static crystallization at 170 °C for 3 d. The resulting gel was vacuum pumped and filtered, washed with deionized water to neutralize, and dried at 100 °C for 30 min. Then, 1 g MCM-49 precursor was dissolved in 50 mL 30 wt% H_2_O_2_ solution in a round-bottomed flask at 90 °C for 12 h to remove the templating agent and then vacuum extraction and drying were performed. The above operations were repeated twice, and the product was calcined at 550 °C for 5 h to obtain Na-MCM-49 zeolite.

### 2.3. The Fabrication of HMCM-49 Zeolite

The Na-MCM-49 zeolite and ammonium nitrate (NH_4_NO_3_) were weighed at a mass ratio of 1:16 and then placed in a 250 mL round-bottomed flask, 100 mL of deionized water was introduced. The mixture was stirred at 90 °C for 10 h, cooled to room temperature naturally and washed with deionized water several times, and then dried. NH_4_-MCM-49 zeolite can be obtained by repeating the above operations twice. The NH_4_-MCM-49 zeolite was calcined in a muffle furnace at 550 °C for 5 h to obtain HMCM-49 zeolite [1].

### 2.4. The Preparation of Mo-Based HMCM-49 Zeolite

HMCM-49 zeolite and commercial MoO_3_ or MoO_3_ nanobelt powder were mechanically mixed according to the mass ratio of 1:0.06, and then calcined in a muffle furnace at 550 °C for 5 h to obtain Mo-based MCM-49, which was respectively recorded as C-Mo-MCM-49 and N-Mo-MCM-49.

### 2.5. Catalyst Characterization

The XRD characterization of all samples was performed on a Smart Lab equipped with Cu Ka radiation (λ = 0.1541 nm; scan speed of 6 min^−1^; 2θ = 5–40°). The morphology and particle size of each sample were tested by FESEM XL-30 field emission scanning electron microscope. Fourier transform infrared spectroscopy (FT-IR) was performed with KBr as a support plate on a Shimadzu Lab Tatal spectrophotometer in the range of 400–4000 cm^−1^. The specific surface and pore size distribution of the samples were recorded by N_2_ adsorption–desorption isotherms at −196 °C on ASAP-2020 equipment. NH_3_-TPD was used to test the acidity of the catalyst on an Auto Chem 2920. Thermogravimetric analysis was performed on Shimadzu DTG-60, the sample was heated to 900 °C at a rate of 5 °C/min. Transmission electron microscopy (TEM) was performed by using a JEM 2100Plus microscopes, the catalyst was dispersed in ethanol using an ultrasonicator and dropped on carbon. H_2_-TPR was performed on an AutoChem 2720 instrument. The sample was treated at 350 °C for 0.5 h in a flow of Ar and cooled down. In the end, the sample was heated. X-ray photoelectron spectra (XPS) of the catalyst was tested with an ESCALAB250XI ray photoelectron spectrometer. Raman analysis of the catalyst was conducted to identify the carbonaceous deposit species of the catalyst. 

### 2.6. Catalytic Tests

The catalytic activity of methane non-aromatization was measured in a fixed-bed reactor equipped with a quartz tube with an inner diameter of 1 cm, in which 0.5 g catalyst (40–60 mesh) was injected. The mass flow meter was used to control the introduction of raw gas (CH_4_ (92.5%) and N_2_ (7.5%)) mixture into the reactor at an airflow rate of 1500 mL g^−1^ h^−1^ using a mass flow controller, and the reaction temperature was controlled at 700 °C. Methane conversion and product yield during the reaction were analyzed using a Tianmei SCION gas chromatograph equipped with a 6 m × 3 mm HayeSep D 80/100 stainless steel column connected to a thermal conductivity detector (TCD) for the analysis of H_2_, N_2_, CO, CH_4_, CO_2_, C_2_H_4_ and C_2_H_6_; the other was a Shimazu CBP1-M50-025 non-polar capillary quartz column to detect benzene, toluene and naphthalene using a hydrogen flame detector. The system used for chromatographic analysis was Lab Solution, and the quantitative basis of methane conversion and hydrocarbon selectivity were calculated based on carbon mass balance using helium as carrier gas and N_2_ as the internal standard [27].

The methane conversion and benzene, toluene, naphthalene, and coke selectivity were calculated according to following mathematical expressions:

$$methane\;conversion\;\left(\%\right)=\frac{F^{in}X_{methane}^{in}-F^{out}X_{methane}^{out}}{F^{in}X_{methane}^{in}}$$

$$S_{product}^{carbon}\;\left(\%\right)=\frac{F^{out}X_{product}^{out}N_{product}^{carbon}}{F^{in}X_{methane}^{in}-F^{out}X_{methane}^{out}}$$

$$S_{coke\;}\left(\%\right)=1-\sum S_{product}^{carbon}$$

*F*, *X* and *N^carbon^* represent total gas flow rate, mole fraction and carbon number in a molecule, respectively.

## 3. Results and Discussion

### 3.1. Structure and Morphology of the Synthesized Catalysts

**Figure 1:**
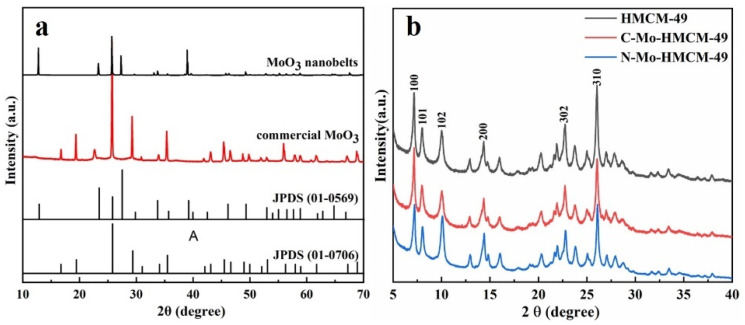
XRD patterns of (**a**) commercial MoO_3_ and MoO_3_ nanobelts, and (**b**) HMCM-49, C-Mo-HMCM-49, and N-Mo-HMCM-49.

XRD reveals the composition and crystal phase structure of the MoO_3_ nanobelts (Figure 1a). As displayed in Figure 1a, the diffraction peaks of commercial MoO_3_ located at 2θ = 12.8°, 23.3°, 25.7°, 27.2°, 38.9° and 67.7° correspond to the (020), (110), (040), (021), (060) and (010) crystal planes, respectively, which is identical to the orthorhombic MoO_3_ (JCPDS card no. 01-0706). All of the peaks over the MoO_3_ nanobelts can be indexed to the hexagonal MoO_3_ ( JCPDS card no. 01-0569) without any impurities, demonstrating that the MoO_3_ nanobelts were successful fabricated [28]. Figure 1b exhibits the XRD patterns of HMCM-49 and HMCM-49 doped with different MoO_3_. The diffraction peaks of HMCM-49 at 2θ = 7.2°, 8.0°, 10°, 14.3°, 22.7°, and 26.0° belong to the (100), (101), (102), (200), (302) and (310) crystal planes, respectively [29]. It can be seen that both catalysts modified with different MoO_3_ present the characteristic diffraction peaks of HMCM-49. Compared with the original HMCM-49, the peak strength of different MoO_3_-modified HMCM-49 decreases, indicating that MoO_3_ has good diffusion in the channel of HMCM-49, resulting in reduced crystallinity. The characteristic peak of the Mo and MoO_x_ species does not appear after loading with MoO_3_, further indicating that the MoO_3_ nanoparticles are well distributed on the surface and channels of HMCM-49. Compared with the C-Mo-HMCM-49 catalyst, the crystallinity of the N-Mo-HMCM-49 samples is lower, especially in the (100) and (310) crystal planes, demonstrating that more Mo species migrate to the channels or surface of HMCM-49 and bind to the acid center in the material, thus reducing the crystallization of zeolite [30].

**Figure 2:**
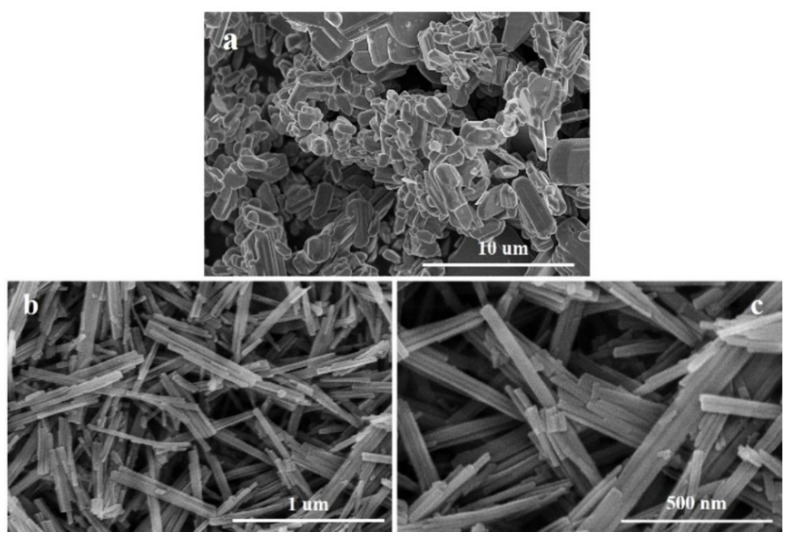
SEM images of commercial MoO_3_ (**a**) and MoO_3_ nanobelts (**b**,**c**).

**Figure 3:**
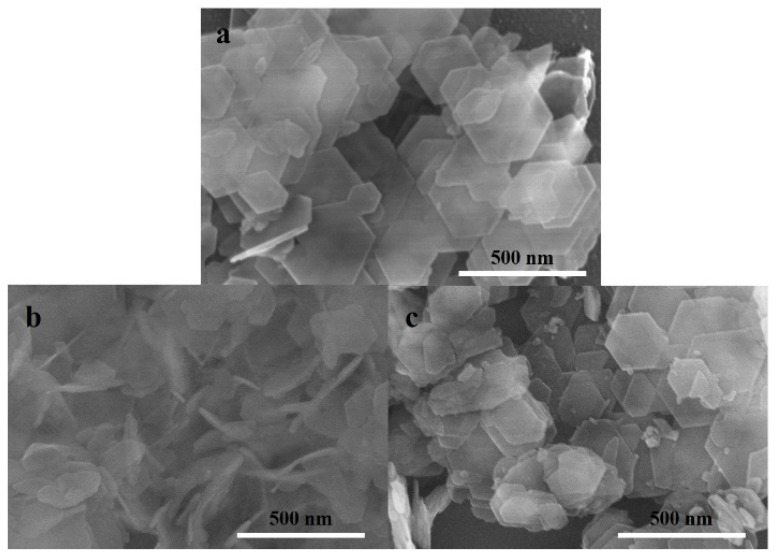
SEM images of (**a**) HMCM-49, (**b**) C-Mo-HMCM-49, and (**c**) N-Mo-HMCM-49.

Figure 2 displays the SEM images of commercial MoO_3_ and MoO_3_ nanobelts. It can be observed that the crystal width of commercial MoO_3_ is about 0.5~0.7 μm and its length is about 0.7~2.1 μm. MoO_3_ nanobelts have a relatively uniform size, smooth surface, and good dispersion. The width of MoO_3_ nanobelts is 15–35 nm and their length is about 120–720 nm. Figure 3 presents the SEM images of the catalyst before and after modification. It can be seen that HMCM-49 is a flake crystal with a regular shape [31]; its length is less than 500 nm and its thickness is less than 50 nm. After the modification of the MoO_3_ nanobelts, the morphology of MCM-49 does not change significantly. However, the crystal particles become smaller on the surface of HMCM-49 after modification with different MoO_3_. This can be attributed to the complete grinding of the HMCM-49 crystal and MoO_3_ nanobelts, or the fact that the large surface area of nano-MoO_3_ bonds easily with the acid center in the zeolite, thus destroying the flake crystal [32]. The above results further indicate that MoO_3_ nanobelts can easily sublimate into the pores of the material after mechanical mixing of MoO_3_ nanobelts and HMCM-49 under high-temperature calcination [33]. Therefore, it is speculated that the MoO_3_ nanobelts can achieve good distribution in the HMCM-49 zeolite without damaging the overall morphology of the crystal.

**Figure 4:**
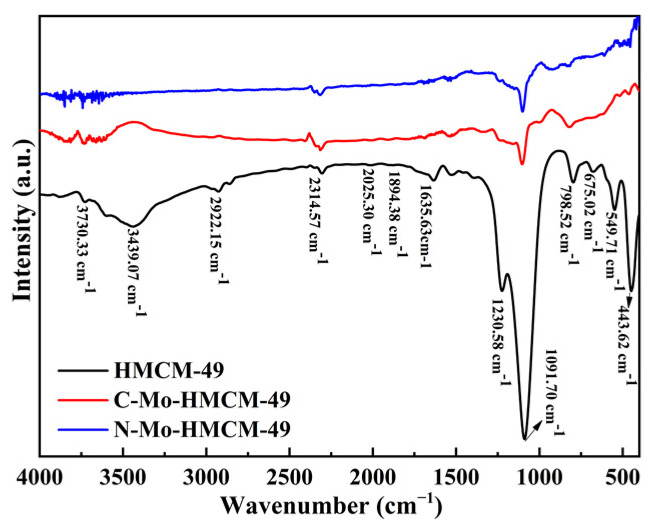
FT-IR spectra of HMCM-49, C-Mo-HMCM-49 and N-Mo-HMCM-49.

The framework structure of the HMCM-49 zeolite was characterized on the basis of infrared spectra. The results are shown in Figure 4. The wide absorption peak of HMCM-49 at 3439.07 cm^−1^ belongs to the Si-OH stretching vibration, and the vibration region lower than 1230.58 cm^−1^ is attributed to the skeleton fundamental frequency vibration region. The absorption peaks at 2025.30, 1894.38 and 1635.63 cm^−1^ are assigned to the co-frequency and pan-frequency bands of the skeleton vibration peak. The bands observed around 1230.58 cm^−1^ and 1091.70 cm^−1^ correspond to Si-O and Al-O asymmetric stretching vibrations, respectively. The absorption peak at 798.52 cm^−1^ is due to Si-O and Al-O symmetric stretching vibration peaks. The absorption peaks at 675.02 and 549.71 cm^−1^ are caused by the double six-ring (D6R) vibration in the HMCM-49 zeolite, and are typical characteristic absorption peaks of microporous zeolite [34]. The peak at 443.62 cm^−1^ is caused by T−O bending vibration in TO_4_ (T is Si or Al), and the above-infrared absorption peak indicates that the synthesized material is HMCM-49 zeolite [35]. Furthermore, it is revealed that when Mo species are loaded onto HMCM-49 zeolite, the position of the sample characteristic absorption peak does not change, and the absorption intensity decreases, indicating that the Mo species have been dispersed into the zeolite channel, which may also damage the material structure to a certain extent.

**Figure 5:**
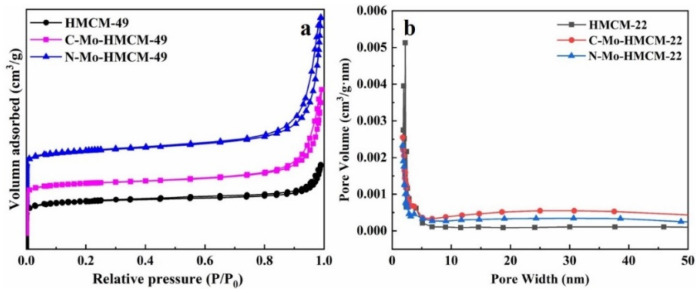
N_2_-adsorption (**a**) and desorption and pore size distributions (**b**) of HMCM-49, C-Mo-HMCM-49 and N-Mo-HMCM-49.

The structures of HMCM-49 and HMCM-49 modified with different MoO_3_ were determined by N_2_ adsorption–desorption isotherms, as shown in Figure 5. It can be observed that all of the samples show typical I-type curves with microporous material characteristics in the region of low specific pressure (P/P_0_ < 0.01), and the adsorption capacity continues to increase in the region with high specific pressure (P/P_0_ = 0.86~1.0), and a small H1-type hysteresis ring appears [18]. This may be caused by the adsorption of N_2_ on the intercrystalline pore and capillary condensation on the crystal surface, indicating that the material contains a certain amount of mesopores. The surface area and pore structure parameters of the material are shown in Table 1. The pore size distributions show that the pore size of the zeolite HMCM-49 after MoO_3_ nanobelt modification increases. In addition, the specific surface area of the material decreases significantly after Mo modification, and the specific surface area of HMCM-49 modified by MoO_3_ nanobelts decreases to 80.6% compared to that of the parent zeolite, indicating that Mo species migrate into the pores of the zeolite and bond with the acid center to promote the dissolution of part of the skeleton of the zeolite, thus increasing the pore size. Meanwhile, MoO_x_ species gather on the surface of the catalyst and block the pore of the zeolite, reducing the specific surface area [36]. The above analysis results indicate that MoO_3_ nanobelts are easy to sublimate and migrate into the pores of HMCM-49 zeolite during the calcination process, resulting in a significant decrease in catalyst surface area [37].

### 3.2. The Surface Acid Studies

**Figure 6:**
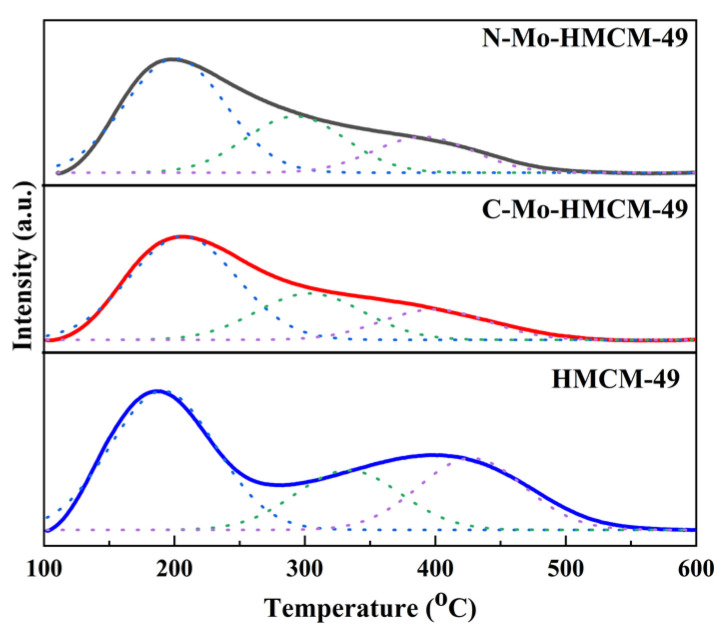
NH_3_-TPD of the catalyst before and after modification.

Acid sites play a vital role in the preparation of excellent MDA catalysts, not only affecting the adsorption of metal sites, but also optimizing the reaction path of aromatic hydrocarbon generation [38]. The surface acidity of the synthesized catalyst was characterized by NH_3_-TPD (Figure 6 and Table 2). There is a new peak after fitting the curve for HMCM-49 and Mo-doped HMCM-49 catalyst. Three NH_3_ desorption peaks are respectively recorded as peak H, peak M and peak L [39]. The center of peak L, located at about 187 °C, is assigned to the physical adsorption of NH_3_ species on Lewis acid and/or NH_3_ stays on non-exchangeable cations, The center of peak M at about 278 °C is attributed to ammonia desorption on the exchangeable protonic acidic sites, and the peak H centered at 417 °C is due to NH_3_ bound to strong Brønsted acid centers [40]. As shown in Figure 6, peak H of the N-Mo-HMCM-49 catalyst shifts at lower temperatures, while peak M increases when HMCM-49 is loaded with different MoO_3_, and peak H of the N-Mo-HMCM-49 catalyst is clearly lower than that of the C-Mo-HMCM-49 catalyst. Combined with the results presented in Table 2, this indicates that Brønsted acid strength decreases significantly. In addition, the N-Mo-HMCM-49 catalyst contributes to the outstanding MDA to a certain extent due to the generation of Mo-O-Al by the interaction of small MoO_3_ with Brønsted acid [41].

**Figure 7:**
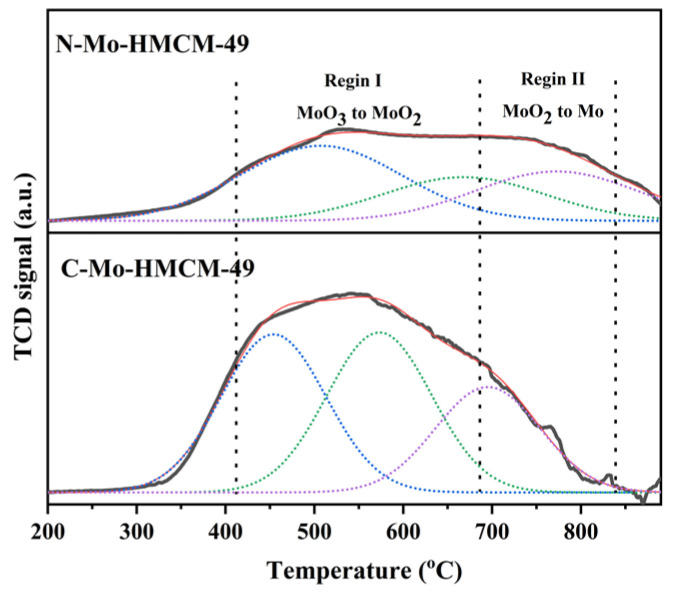
H_2_-TPR profiles of the C-Mo-HMCM-49 and N-Mo-HMCM-49 catalysts.

H_2_-TPR profiles of the C-Mo-HMCM-49 and N-Mo-HMCM-49 catalysts were determined in order to distinguish the different Mo species in the samples and their degree of reducibility (Figure 7). The different reduction features of MoO_x_ species in Mo-based HZSM-5 zeolite have been reported, and the reduction located at 370–580 °C can be ascribed to the reduction of MoO_3_ to MoO_2_, whereas the reduction of MoO_2_ to Mo occurs at higher temperatures—above 600 °C [42]. It can be observed that when moving from N-Mo-HMCM-49 to C-Mo-HMCM-49, the reducibility of initial MoO_3_ to MoO_2_ in N-Mo-HMCM-49 catalyst increases, which can be considered to be due to the change in the anchoring sites of MoO_3_ species inside the HMCM-49 channels with respect to different kinds of MoO_3_ loading. Lower reducibility over N-Mo-HMCM-49 can be observed, which is due to the strong interaction of MoO_x_ species with HMCM-49 [2]. Therefore, the variation in the reducibility of MoO_x_ species, as shown in the H_2_-TPR analysis, verifies that the MoO_3_ species change in the HMCM-49 zeolite significantly influences the binding of MoO_x_ species.

**Figure 8:**
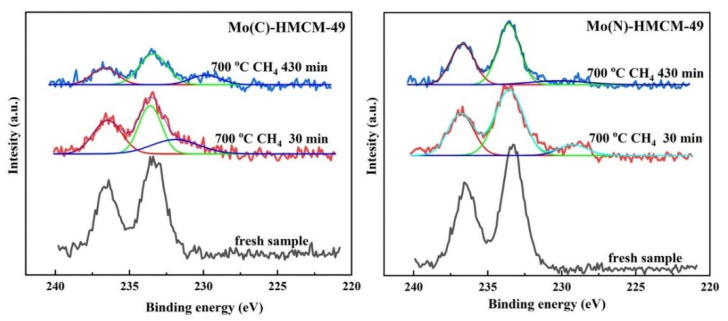
Mo 3d region XPS spectra of the C-Mo-HMCM-49 and N-Mo-HMCM-49 catalysts before and after reaction under CH_4_ flow at 700 °C for 30 and 430 min.

**Figure 9:**
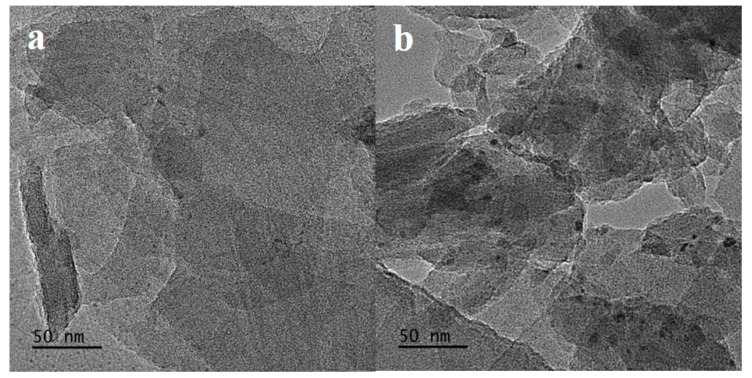
TEM image: (**a**) the catalyst N-Mo-HMCM-49 before reaction and (**b**) the catalyst N-Mo-HMCM-49 after the reaction under CH_4_ flow at 700 °C for 430 min.

The Mo 3d region XPS spectra of the C-Mo-HMCM-49 and N-Mo-HMCM-49 catalysts before and after reaction under CH_4_ flow at 700 °C for 30 and 430 min are shown in Figure 8. Molybdenum carbide (Mo_2_C) dispersed in zeolite channels is the active site of C-H bond activation, and is formed during the induction stage of the MDA reaction process [5,43]. It can be observed that the Mo 3d binding energies at 236.6 and 233.4 eV can respectively be assigned to the Mo^6+^(3d_3/2_) and Mo^6+^(3d_5/2_) molybdenum oxide species for fresh catalysts, whereas the binding energy at 228.9 eV can be attributed to the molybdenum carbide species (Mo_2_C) formed for the catalysts after the reaction under CH_4_ flow at 700 °C for 30 and 430 min. This can be clarified by the dispersion of Mo oxidation state, which can be observed to be 24.8% (Mo_2_C), 39.8% (Mo^6+^, 3d_5/2_) and 35.4% (Mo^6+^, 3d_3/2_) for N-Mo-HMCM-49 catalyst after the reaction under CH_4_ flow at 700 °C for 30 min, and 18.1% (Mo_2_C), 54.4% (Mo^6+^, 3d_5/2_) and 27.5% (Mo^6+^, 3d_3/2_) for N-Mo-HMCM-49 catalyst after the reaction under CH_4_ flow at 700 °C for 430 min, whereas for C-Mo-HMCM-49, the distribution observed is 9.5%/8.7% (Mo_2_C), 57.4%/53.3% (Mo^6+^, 3d_5/2_) and 33.1%/33.1% (Mo^6+^, 3d_3/2_). It is clear that the proportion of observed Mo_2_C species is significantly higher for the N-Mo-HMCM-49 catalyst, which indicates a higher degree of carburization. Furthermore, the sample of N-Mo-HMCM-49 clearly exhibits good dispersion of carbine clusters (MoC_x_) after the reaction under CH_4_ flow at 700 °C for 430 min, as shown in Figure 9. Gao et al. demonstrated different molybdenum carbide (Mo_x_C_y_) structures and their anchoring inside ZSM-5 channels on the basis of theoretical studies [44]. The results of the XPS and TEM analysis clearly confirm that the higher degree of carburization for N-Mo-HMCM-49 can be ascribed to the higher concentration of MoO_x_ species anchored inside the channel of HMCM-49.

**Figure 10:**
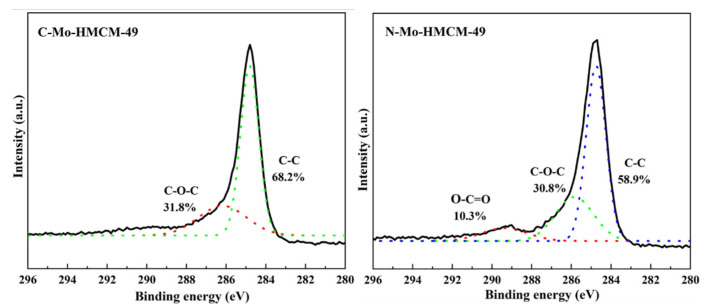
C 1s region XPS spectra of the C-Mo-HMCM-49 and N-Mo-HMCM-49 catalysts after reaction under CH_4_ flow at 700 °C for 430 min.

C 1s region XPS spectra of the C-Mo-HMCM-49 and N-Mo-HMCM-49 catalysts after reaction under CH_4_ flow at 700 °C for 430 min confirmed that the C-Mo-HMCM-49 catalyst has greater carbon deposition. As shown in Figure 10, the C-C bond due to hard coke formed by polyaromatic or pre-graphitic species at strong Brønsted acid sites, while the C-O-C and O-C=O bonds are ascribed to the graphitic coke formed at the oxycarbide active sites (MoO_x_C_y_) [45]. These results demonstrate that the higher Mo distribution in the case of the N-Mo-HMCM-49 sample, which also inhibits the formation of hard coke, maybe conducive to a different catalyst deactivation mechanism.

**Figure 11:**
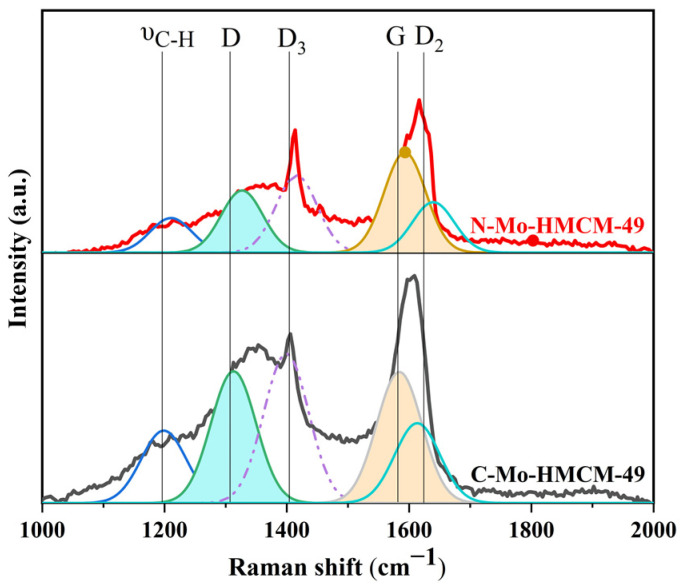
Raman spectra of the C-Mo-HMCM-49 and N-Mo-HMCM-49 catalysts after reaction under CH_4_ flow at 700 °C for 430 min.

The catalysts of C-Mo-HMCM-49 and N-Mo-HMCM-49 after reaction under CH_4_ flow at 700 °C for 430 min have almost identical Raman spectra, as shown in Figure 11. They exhibit five-peak-based deconvolution of the intense signals associated with carbon species. It is clear that the Raman shift located at 1200 cm^−1^ can be ascribed to aliphatic C-H bounds, the Raman shift around 1312 cm^−1^ represents the D band, which is attributed to the aromatic structures or amorphous coke, the D_3_ band around 1405 cm^−1^ is due to structural defects of aromatic domains with poor organization, and the G band located at 1590 cm^−1^ is assigned to graphite-like coke while D_2_ belongs to the D echo band. It can be clearly seen that the ratio between D and G band intensities is lower for N-Mo-HMCM-49 (I_D_/l_G_ = 0.62) than that of C-Mo-HMCM-49 (I_D_/l_G_ = 1.01), demonstrating that the degree of the disordered nature of the surface carbon species is more remarkable for C-Mo-HMCM-49 [46]. Furthermore, the coke produced from MoC_x_ is well known to be amorphous, which promotes the deactivation of the catalyst [47,48]. Meanwhile, HMCM-49 modified with MoO_3_ nanobelts may prevent excessive coke formation on MoC_x_ and would improve catalytic stability.

### 3.3. Catalytic Performance

**Figure 12:**
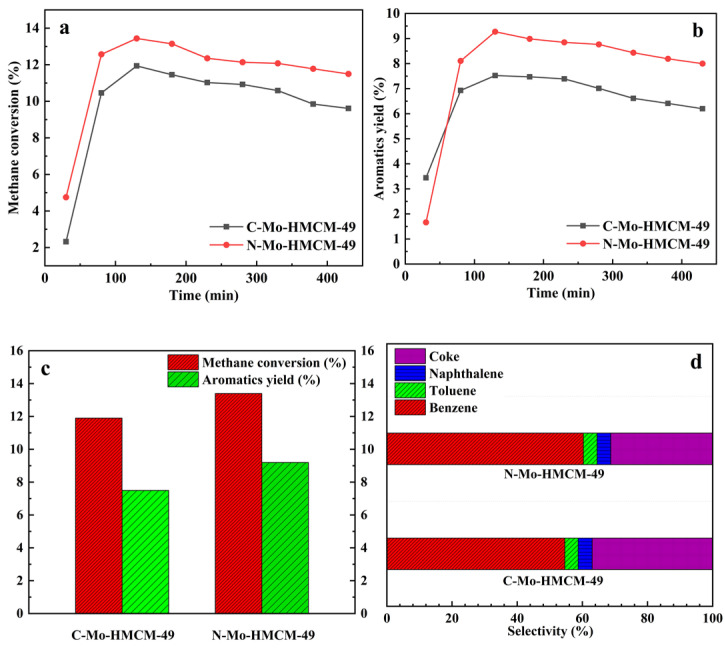
Catalytic performance of MDA over C-Mo-HMCM-49 and N-Mo-HMCM-49: (**a**) methane conversion, (**b**) aromatics yield, (**c**) the comparison of catalyst performance over both catalysts and (**d**) the selectivity of various products. Reaction conditions: T (temperature) = 700 °C, P (pressure) = 1 atm, GHSV (gas firing hourly space velocity) = 1500 h^−1^.

**Figure 13:**
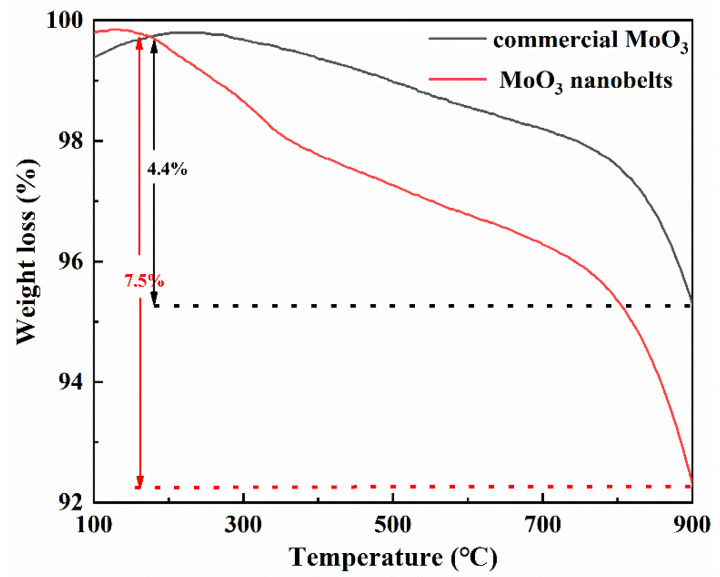
Thermogravimetric analysis of commercial MoO_3_ and MoO_3_ nanobelts.

Figure 12 and Table 3 display the methane conversion, aromatics yield, and selectivity of different products (benzene, toluene, naphthalene and coke) over C-Mo-HMCM-49 and N-Mo-HMCM-49 in MDA [49]. It can be clearly observed from Figure 12a that the methane conversion rate demonstrates an increasing trend during the initial reaction stage of less than 130 min, implying the existence of an induction period for the MDA reaction during which active site molybdenum carbide species (Mo_2_C or MoO_x_C_y_) are generated [50]. The highest methane conversion rates of C-Mo-HMCM-49 and N-Mo-HMCM-49 are 11.9% and 13.4%, respectively, and the corresponding aromatic hydrocarbon yields are 7.5% and 9.2% (Figure 12b). Furthermore, the value TOF of 15.1 h^−1^ over N-Mo-HMCM-49 is higher than that of C-Mo-HMCM-49 (14.2 h^−1^). Compared to the 6Mo(N)-MCM-22 catalyst previously reported, N-Mo-HMCM-49 exhibits a better TOF value. It is worth noting that the methane conversion rate for all catalysts continues to decline with increasing time on stream (TOS), meaning that catalyst inactivation occurs continuously during the reaction, which may be attributed to the sintering and separation of carbon and metal carbonization species accumulated on the zeolite [51]. Additionally, it can be clearly seen from Table 3 that 11.5% methane conversion is obtained on N-Mo-HMCM-49, which exceeds that (9.8%) on C-Mo-HMCM-49 after 430 min, and the aromatics yields of C-Mo-HMCM-49 and N-Mo-HMCM-49 are respectively 6.2% and 8.0% at this point. The MCM-49 zeolite has a set of 12-ring super cages and 10-ring channels connected through 10-ring windows. The unique pore systems are beneficial for benzene and aromatics formation. Mo carbides located on the surface of zeolite mainly generate heavy hydrocarbon and carbon deposition, leading to a decrease in methane conversion and aromatic hydrocarbon yield [51]. Furthermore, as illustrated in Figure 13, the weight loss of MoO_3_ nanobelts is 7.5% below 900 °C, which is higher than the weight loss (4.4%) of commercial MoO_3_. Thermogravimetric analysis shows that MoO_3_ nanobelts sublimate more easily than commercial MoO_3_. The improvement in catalytic activity may be related to the good dispersibility of active Mo sites. It is likely that the structure of Mo precursors plays a significant role in catalytic performance. Mo precursors supported on carrier material have multiple structures, which results in complex and widened signals for most spectroscopic techniques [1]. Combined with the results of acid tests, MoO_3_ nanobelts can be effectively transferred to MCM-49 channels by regulating the acidity distribution and interacting with strong acid sites to generate more effective active Mo sites and achieve better catalytic performance [30].

Figure 12d indicates the selectivity of various products for both Mo-based catalysts. In combination with Table 3, it can be seen that N-Mo-HMCM-49 has higher benzene selectivity (60.3%) than C-Mo-HMCM-49 (54.6%) after reaction for 130 min, and the corresponding coke selectivities are 37.0% and 31.4%, respectively. The improved selectivity of benzene modified by MoO_3_ nanobelts may be partly due to the strong inhibition of coking by external Brønsted sites. However, the deposition of carbonaceous species eventually blocks the zeolite channel, making it difficult for products to diffuse out of the zeolite channel [52]. Combined with the results of Raman spectra, HMCM-49 modified with MoO_3_ nanobelts would inhibit excessive coke deposition and thus improve catalytic stability.

**Figure 14:**
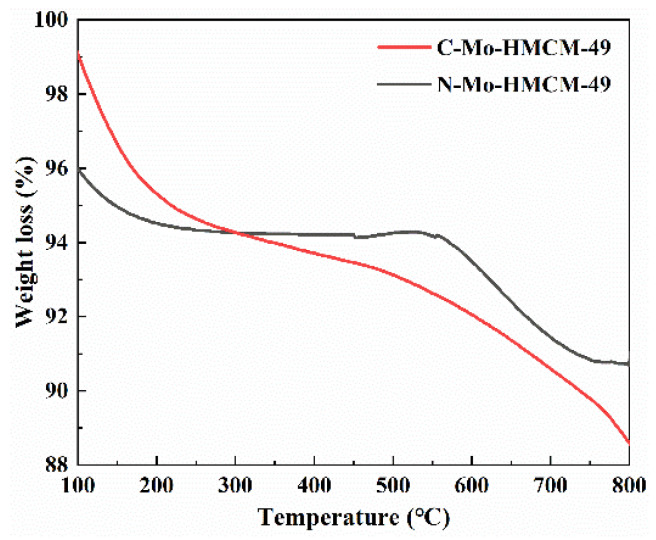
TG curves of C-Mo-HMCM-49 and N-Mo-HMCM-49 reaction for 430 min.

As shown in Figure 14, the weight loss below 220 °C is due to the evaporation of water. The weight loss between 220 and 400 °C is caused by the aromatics of physical absorption, and it is noteworthy that the carbon deposits on zeolite are burned and caused weight loss in the range of 400 to 800 °C [53]. Benzene is mainly formed in the channels of the zeolite, whereas naphthalene is produced on the external surface and/or the pore mouth of the zeolite [1,54]. Carbon deposition in the zeolite channel will not only block the zeolite channel, but also poison the catalytically active center, eventually leading to catalyst inactivation [52]. The weight-loss rates of C-Mo-MCM-49 and N-Mo-MCM-49 after 430 min are 5.76% and 3.47%, respectively, indicating that N-Mo-MCM-49 has better anti-carbon deposition ability than C-Mo-MCM-49 [55]. Consequently, N-Mo-MCM-49 shows better catalytic stabilization in MDA reactions.

## 4. Conclusions

In summary, MCM-49 zeolite modified with MoO_3_ nanobelts exhibits better MDA performance than commercial MoO_3_-modified zeolite. XRD, FT-IR, N_2_ adsorption/desorption NH_3_-TPD, XPS, TEM, H_2_-TPR and Raman spectra indicate that MoO_3_ nanobelts are easy to sublimate and distribute into the channel of HMCM-49 zeolite and combine with the strong acid center located on the zeolites’ inner surface to form the active Mo site, thus promoting methane activation, inhibiting the coke deposition formed by external Brønsted sites, and increasing the yield of aromatics. The methane conversion rate of MoO_3_ nanobelt-modified HMCM-49 can reach 13.4%, and the corresponding aromatic hydrocarbon yield can reach 9.2%. This work lays the groundwork for the usage of nano-metal precursors to develop new catalysts and further enhance the MDA performance.

## Figures and Tables

**Table 1:** The textural properties of HMCM-49, C-Mo-HMCM-49, and N-Mo-HMCM-49.

Samples	S_BET_^a^	S_micropore_^b^ (m^2^/g)	S_external_^b^ (m^2^/g)	V_micropore_^b^ (cm^3^/g)	V_total_^c^ (cm^3^/g)	Pore Size (nm) ^d^
HMCM-49	382	312	70	0.14	0.26	8.2
C-Mo-HMCM-49	327	238	93	0.11	0.54	16.8
N-Mo-HMCM-49	308	233	70	0.11	0.49	16.6

^a^ Calculated using the BET method. ^b^ Calculated by the t-plot method. ^c^ Calculated from the adsorption capacity at P/P_0_ of 0.98. ^d^ BJH Desorption average pore width (4V/A).

**Table 2:** The textural properties of HMCM-49, C-Mo-HMCM-49 and N-Mo-HMCM-49.

Samples	Peak Temperature (°C)			H_2_ Consumption (µmol·g^−1^)			Total
	Peak 1	Peak 2	Peak 3	Peak 1	Peak 2	Peak 3	
HMCM-49	191	333	427	348	178	187	713
C-Mo-HMCM-49	207	304	398	268	121	80	469
N-Mo-HMCM-49	201	293	391	254	125	78	457

**Table 3:** The catalytic results of MDA over various catalysts.

Catalyst	ReactionTime (min)	Conversion of CH_4_ (%)	TOF (h^−1^)	Selectivity (%)				Yields of Aromatics (%)
				Benzene	Toluene	Naphthalene	Coke	
C-Mo-HMCM-49	130	11.9	14.2	54.6	4.1	4.3	37.0	7.5
	430	9.8	11.7	56.7	4.0	4.5	34.8	6.2
N-Mo-HMCM-49	130	13.4	15.1	60.3	4.2	4.1	31.4	9.2
	430	11.5	12.9	62.3	4.0	3.9	29.8	8.0
6Mo(N)-MCM-22 [48]	130	13.1	14.8	61.1	3.1	4.2	31.6	8.9
	580	10.4	11.7	62.5	3.9	3.8	29.4	7.3

## Data Availability

Not applicable.
